# Molecular features in arsenic-induced lung tumors

**DOI:** 10.1186/1476-4598-12-20

**Published:** 2013-03-19

**Authors:** Roland Hubaux, Daiana D Becker-Santos, Katey SS Enfield, David Rowbotham, Stephen Lam, Wan L Lam, Victor D Martinez

**Affiliations:** 1 British Columbia Cancer Research Centre, 675 West 10th Avenue, Vancouver, BC V5Z 1L3, Canada

**Keywords:** Arsenic, Arsenite, Lung cancer, Epigenetic, Reactive oxygen species, Epidermal growth factor receptor, Phosphatidylinositol 3-kinases, NFE2-related factor 2

## Abstract

Arsenic is a well-known human carcinogen, which potentially affects ~160 million people worldwide via exposure to unsafe levels in drinking water. Lungs are one of the main target organs for arsenic-related carcinogenesis. These tumors exhibit particular features, such as squamous cell-type specificity and high incidence among never smokers. Arsenic-induced malignant transformation is mainly related to the biotransformation process intended for the metabolic clearing of the carcinogen, which results in specific genetic and epigenetic alterations that ultimately affect key pathways in lung carcinogenesis. Based on this, lung tumors induced by arsenic exposure could be considered an additional subtype of lung cancer, especially in the case of never-smokers, where arsenic is a known etiological agent. In this article, we review the current knowledge on the various mechanisms of arsenic carcinogenicity and the specific roles of this metalloid in signaling pathways leading to lung cancer.

## Introduction

**Figure 1:**
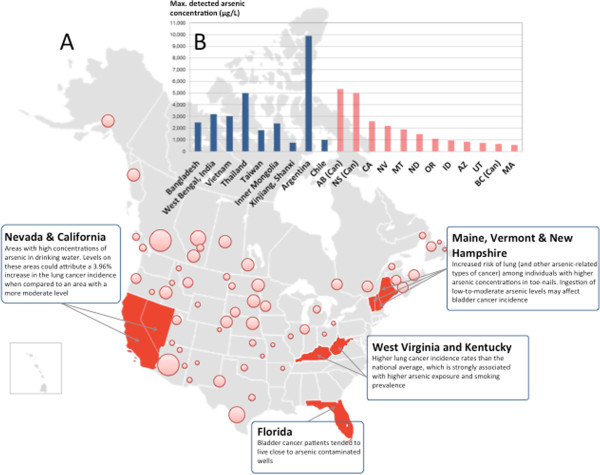
**Arsenic occurrence in North America and documented relationship with different cancer types. A**) Light red circles on the map represent Canada provinces and US States affected by arsenic concentrations over 10μg/L. Estimations were made on the basis of data obtained from Health Canada [10] and the US Geological Service [11]. Please note that circled areas are approximations only; for detailed information, see references. States in red indicate evidence of arsenic exposure and higher cancer incidence, based on published literature. **B**) Histogram representing maximum concentrations detected in countries with evidence of arsenic exposure and cancer relationship (blue) [12] and in provinces/states of Canada and United States (light red) [10,11].

Arsenic is a well-known human carcinogen [1]. This metalloid is widely distributed throughout the Earth’s crust and arsenical species tend to remain in solution even at high concentrations (tens of μg/L) at near-neutral pH [2]. As a result, arsenic exposure through drinking water is considered the cause of the largest mass poisoning worldwide. In Bangladesh, more than 70 million people are at risk of long term exposure to high levels of arsenic through groundwater [3]. On the other hand, chronic exposure to low-levels of arsenic in drinking water is an emerging risk across different parts of the world, including North America (Figure 1) [4-7]. Paradoxically, arsenic (as arsenic trioxide, A_2_O_3_) is also used as therapeutic agent in the treatment of acute promyelocytic leukemia [8,9].

Common types of tumors associated with arsenic exposure are found in skin, bladder, liver and lung. Following arsenic exposure, lung cancer has proven to be amongst the most deadly cancer types [13,14]. Lung adenocarcinoma is the most common type of lung cancer worldwide, however, the most frequent histological subtypes observed in arsenic-induced lung tumors - among both smokers and non-smokers - are squamous cell carcinomas (SqCC) and small cell carcinomas (SCC) [15]. Lung tumors derived from individuals exposed to arsenic also exhibit differential genetic and epigenetic changes when compared to histologically matched tumors derived from an arsenic-free environment. The differential molecular alterations seen in arsenic-induced tumors may not arise from inorganic arsenic, but instead from more damaging arsenic species generated through its metabolism [16]. In this article, we discuss mechanisms that enhance the carcinogenic potential of arsenic, such as its biotransformation, as well as the impact of this carcinogen and its derivatives at a molecular pathway level.

### Molecular mechanisms involved in arsenic-induced carcinogenesis

**Figure 2:**
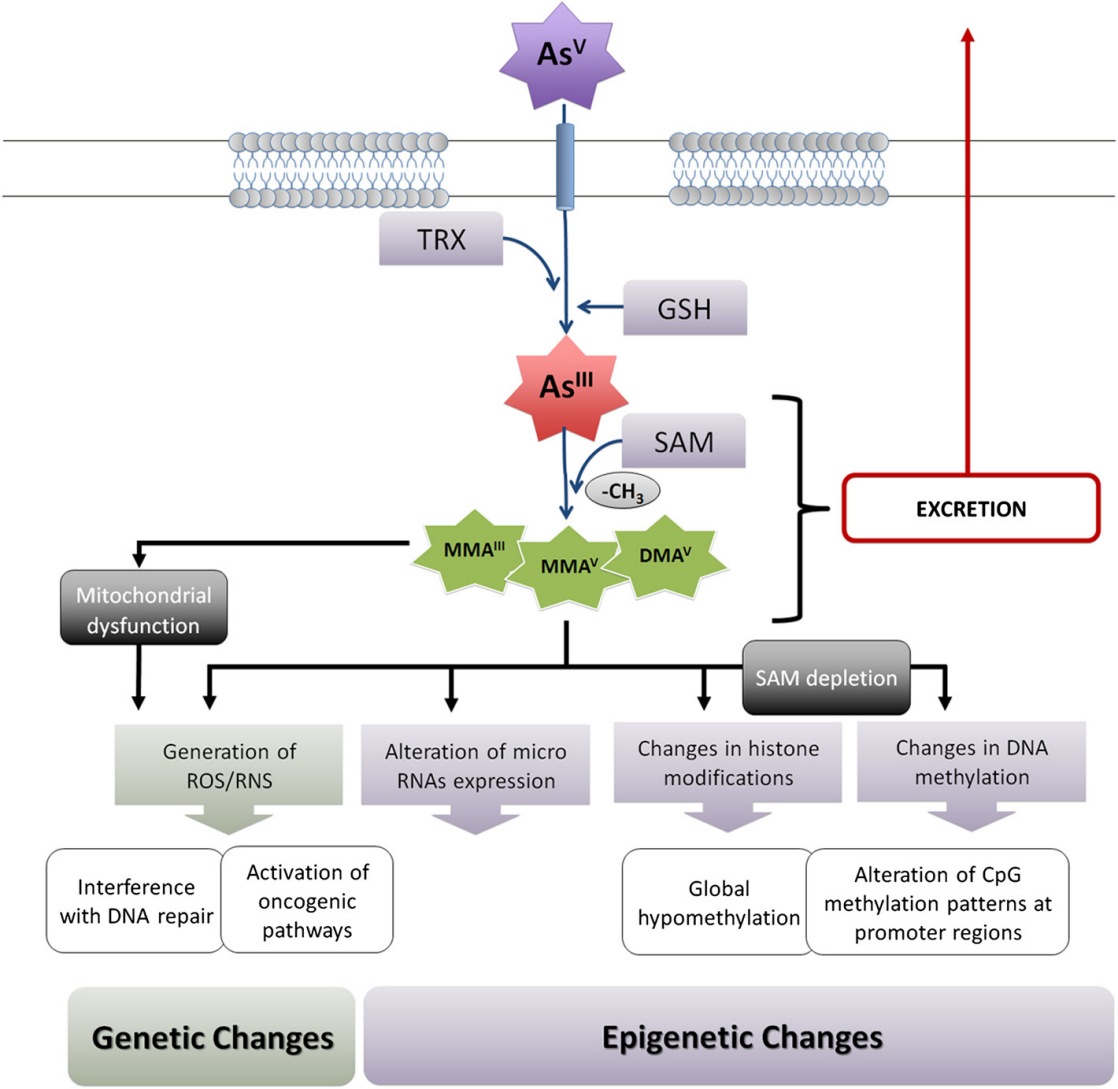
**Mechanisms of arsenic-induced carcinogenesis. **Carcinogenic effects induced by arsenic exposure are mostly generated due to its biotransformation process, having effects at genetic and epigenetic levels. Arsenic biotransformation occurs through a series of cycles of reduction, oxidation, and methylation reactions. Pentavalent arsenic (As^V^) is reduced to arsenite (As^III^), using glutathione (GSH) and thioredoxin (TRX) as electron donors. In the excretion process, As^III ^is methylated using S-Adenosyl methionine (SAM) as a source of methyl groups resulting in generation of arsenic species with higher carcinogenic potential. Genetic alterations are largely due to generation of reactive oxygen and/or nitrogen species, partially derived from arsenic-induced mitochondrial dysfunction. Epigenetic effects, such as changes in DNA methylation patterns have been linked to deprivation of SAM.

The carcinogenic capacity of arsenic is causally linked to its biotransformation (Figure 2) [17]. Inorganic arsenic is readily absorbed by the gastrointestinal tract when ingested through drinking water [18]. Upon ingestion, arsenic is predominantly found in its pentavalent form (arsenate, As^v^) and enters cells through membrane transporters such as inorganic phosphate transporters (PiT) and aquaporins [19,20]. Inside the cell, As^V^ is reduced to the more toxic arsenite (As^III^) in a glutathione-dependent reaction driven by polynucleotide phosphorylase and mitochondrial ATP synthase [21]. As a part of a cellular detoxification process, As^III^ and its methylated conjugates are translocated from hepatocytes into bile as glutathione conjugates [22]. Mono- and dimethylated As^III^ species leaving the liver are highly reactive and have been shown to induce damage in different organs, including lungs. This damage occurs primarily through the generation of reactive oxygen species (ROS) in concert with glutathione depletion [23-25]. Increased toxicity of As^III^ can be attributed to a high covalent reactivity towards thiol groups; thus, the metalloid often interacts with proteins to induce their inactivation/degradation [20].

### Arsenical species induce genetic alterations

#### Arsenic as a co-mutagen

Inorganic arsenic does not interact directly with DNA and is not considered to be mutagenic at non-toxic doses [26]. However, as previously described, methylated arsenic species and other byproducts generated in the biotransformation process are potent clastogens and mutagens [27,28]. Furthermore, low doses of arsenic can potentiate mutagenic effects through other carcinogens such as UV light, N-methyl-N-nitrosourea, diepoxybutane, X-rays, methylmethane sulfonate and tobacco [29-34].

### Arsenic induces DNA damage via generation of reactive oxygen and nitrogen species

Arsenic-induced ROS may be generated by either cycling of As^III^ and As^V^[35] or through disruption of the mitochondrial electron transport chain [36] (Figure 2). Most of the known arsenic-related mechanisms of ROS generation involve the latter mechanism. Typically, mitochondrial ROS is generated through monomethylarsonous acid (MMA^III^)-mediated inhibition of mitochondrial complexes II and IV [16], which results in a back-log of electrons and, eventually, electron leakage from complexes I and III [37]. Liberation of electrons from the electron transport chain (ETC) leads to formation of superoxide anion radicals (O2•^-^), hydrogen peroxide (H_2_O_2_), and hydroxyl radicals (OH•) [19,38]. Arsenic-mediated production of free-radical species has been associated with the formation of DNA adducts, DNA double-stranded breaks, DNA cross linking, chromosomal aberrations, DNA mutations and DNA deletions (Figure 2) [39-41].

Arsenic can also induce generation of reactive nitrogen species (RNS). The mechanisms involved are not completely understood; however, they are thought to occur in a tissue-specific manner [42]. The increase in amounts of RNS such as peroxynitrite has been shown to cause DNA alkylation, deamination, and oxidative DNA damage [43-47].

### Arsenic interferes with DNA repair processes

Arsenic can affect cellular DNA repair capacity, by altering both nucleotide- (NER) and base-excision repair (BER) mechanisms (Figure 2). Arsenic interferes with NER by reducing the frequency of incision steps of the repair process [30], reducing the expression of NER-associated genes and decreasing expression and protein levels of Xeroderma pigmentosum complementation group C (XPC) [48-50]. In addition, methylated As^III^ species generated by the biotransformation process impair the expression and activity of human PARP1, a promoter of NER that acts in response to DNA damage [51]. Arsenic metabolites also decrease gene expression and protein levels of BER-related components, such as 8-oxoguanine DNA glycosylase 1 (hOGG1), DNA ligase IIIα (LIGIIIα), and X-ray cross complementing protein 1 (XRCC1) [17]. In arsenic-exposed murine lung tissue, the expression of several genes related to BER - such as apurinic/apyrimidinic, endonuclease/redox effector-1 (APE1), ligase I, DNA, ATP-dependent (LIG1), 8-oxoguanine DNA glycosylase (OGG1), and poly (ADP-ribose) polymerase 1 (PARP1) - were elevated [52].

### Arsenic induces chromosomal and genomic instability

Arsenic-treated cells demonstrate significantly increased micronuclei formation as well as chromosomal aneuploidy, likely by an effect on sulfhydryl groups of tubulin and microtubule-associated proteins and consequential cell spindle assembly disruption [53-57]. Additional studies have shown that the p53-dependent increase in p21 expression observed in normal cells following DNA damage is inhibited in cells exposed to arsenic, leading to cell cycle progression despite heavy DNA damage and genomic instability [58-61]. Similarly, arsenic-induced disruption of PARP1 activity contributes to genomic instability by allowing the survival of cells with significant DNA lesions [51,62]. Studies comparing DNA copy number alterations in arsenic-exposed and non-exposed lung tumor cells indicate the location and frequency of alterations differ between the two cases. Genomes of lung tumors from patients who never smoke, as well as those chronically exposed to arsenic harbor segmental DNA amplifications at 19q13.31 and 19q13.33 and segmental DNA losses at chromosomal locus 1q21, among others [63,64]. Interestingly, genes in 19q13.33, such as Spleen focus forming virus (SFFV), proviral integration oncogene B (SPIB), and Nuclear receptor subfamily 1, group H, member 2 (NR1H2) have been shown to be oncogenic in mouse models [65-67].

### Arsenic-induced epigenetic alterations

#### Arsenic biotransformation depletes SAM resulting in aberrant DNA methylation

Arsenic detoxification requires the use of S-Adenosyl methionine (SAM) as a methyl donor (Figure 2); consequently, arsenic-related epigenetic effects mainly derive from deprivation of the cellular pool of methyl (-CH3) groups [68]. Although cellular levels of SAM itself are not likely affected, a high demand of SAM due to chronic arsenic exposure will affect the availability of the cellular pool of methyl groups [69-71]. Since SAM is the major methyl donor for DNA-methyltransferases (DNMT), depletion of methyl groups can lead to global hypomethylation and changes in chromatin remodeling [72,73]. Such epigenetic modifications have been shown to promote malignant transformation in a variety of cell types, including lung [74-76]. Arsenic has been shown to induce global hypomethylation, as demonstrated by reduction in LINE-1 methylation and total 5-methyldeoxycytidine content in lymphoblastoid cells [72]. Importantly, even low-level arsenic exposure resulted in DNA hypomethylation in rat models [77]. Moreover, arsenic-induced SAM deprivation can alter CpG methylation status of promoters for specific genes, such as Deleted In Bladder Cancer 1 (DBC1), Death-Associated Protein Kinase 1 (DAPK), and P53 [68,78-86]. ROS generated during arsenic biotransformation can also interfere with DNA methylation and contribute to aberrant epigenetic modifications and deregulation of gene expression [87].

Interestingly, individuals chronically exposed to high yet non-lethal levels of arsenic exhibit a significantly higher degree of DNA methylation in promoter regions of P53 and CDKN2A compared to non-exposed controls [88]. Lung cancer cell models have also shown that arsenic exposure resulted in P53 promoter hypermethylation and subsequent transcriptional silencing of this gene [78]. Promoter hypermethylation of tumor suppressors CDKN2A and RASSF1A was also observed in lung tumors of mice exposed to inorganic arsenate [75].

### Arsenic changes gene expression patterns by altering histone modification

Arsenic-mediated reduction of global levels of H4K16 acetylation, a mark of gene activation, has been demonstrated in cell models [89]. Further, arsenic exposure has been shown to modify H3K4, H3K9, and H3K27 histone methylation patterns in both malignant and non-malignant lung cell lines, leading to a decrease in the expression of genes associated with histone acetylation and DNA methylation changes [80,90]. Arsenic has also been reported to alter the chromatin landscape of arsenic-induced cancer cells through loss of the repressive histone modifications H3 triMe-K27 and H3 diMe-K9 and an increase in the levels of activating Ac-H3 and diMe-K4 at the WNT5A locus - resulting in the ectopic expression of WNT family genes [73].

### Arsenic induces epithelial-to-mesenchymal transition and other biological effects through changes in micro-RNA expression

A study using human bronchial epithelial cells (HBEC) demonstrated that chronic arsenic exposure of P53-knock down cells induced malignant transformation accompanied by epithelial-to-mesenchymal transition (EMT) [91]. A reduction in expression of a miR-200 family member was correlated with this exposure, and was shown to occur through increased promoter methylation. Re-establishment of miR-200b expression alone was capable of entirely reversing and preventing arsenic-induced EMT and malignant transformation [91].

Arsenic exposure can alter miRNA expression levels in vitro and in vivo in other cell types and tissues. For example, in a study using chick embryos, arsenic decreaseD expression of miR-9, -181b, -124, and -125b. Decrease of miR-9 and miR-181b resulted in expression of their common target Nrp1, leading to cell migration, tube formation and angiogenesis [92]. Arsenite induced overexpression of several miRNAs, including miR-222, in human peripheral blood-derived cells from individuals with insufficient dietary folate. Overexpression of miR-222 was reversed by the restoration of normal folate levels [76].

### Arsenic targets key pathways associated with lung cancer

#### Arsenic stimulates the EGFR signaling pathway

Alteration in the EGFR pathway can result from mutation and/or amplification events at the epidermal growth factor receptor (EGFR) locus. The consequence of either genetic event is a structural alteration that destabilizes the auto-inhibitory loop of EGFR, forcing the receptor into a constitutive and ligand-independent active state [93].

**Figure 3:**
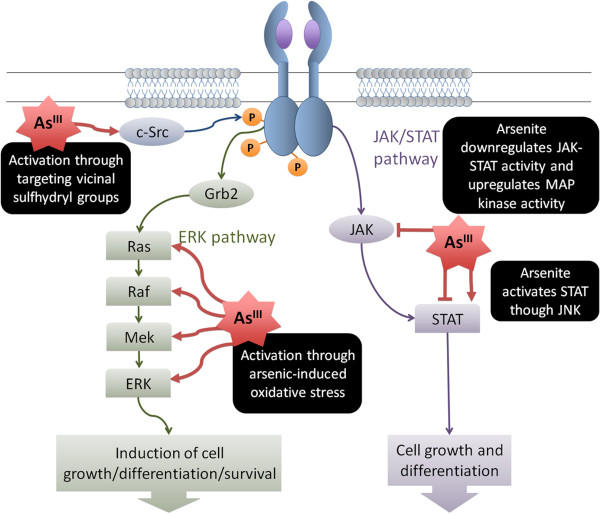
**Arsenic-mediated activation of EGFR signaling pathway. **EGFR and several components of this pathway can be activated by arsenic exposure in human lung cells. This activation can be inhibited by EGFR-TKI, revealing a potential role for TKIs in the management of arsenic associated lung tumors, regardless of the mutational status of EGFR. As^III ^can also induce STAT3 inhibition by targeting JAK, while it can activate STAT3 trough JNK, contributing to AKT activation.

Similar states of EGFR constitutive activation can be induced by even moderate levels of arsenic, similar to those registered in contaminated U.S. drinking water, affecting the lungs and other target organs of arsenic carcinogenesis [94,95] (Figure 3). Arsenic can stimulate c-Src activity, which can then activate EGFR by physical interaction resulting in two unique tyrosine phosphorylation events (Tyr845, Tyr1101), leading to ligand-independent EGFR phosphorylation and constitutive activation [96-98]. Arsenic can also induce activation of components of the EGFR pathway in lung epithelial cells, such as Ras, Raf, Mek and ERK through ROS [94,99,100]. Arsenite inhibits STAT3 through JAK inactivation, and such interference may play a role in arsenic-associated pathogenesis [101]. Conversely, it has been shown that As^III^ activates STAT3 through c-Jun NH2 kinase (JNK), contributing to Akt activation [102]. Arsenic-exposed hepatocellular carcinoma cells display overexpression of EGFR [95], while in leukemia cell lines, As^III^ is capable of activating Rac1 GTPases resulting in downstream engagement of the JNK pathway and increased cell survival and proliferation [103,104]. This arsenic-related induction of EGFR signaling offers promising therapeutic utility, as inhibitors of EGFR and various other pathway components are already in place or in development [105].

### Arsenic and the PI3K/AKT signaling pathway

Signaling through the PI3K/AKT pathway starts with the activation of receptor tyrosine kinases (RTK’s) through binding to an extracellular growth factor. Binding of the extracellular ligand to its receptor leads to the dimerization and activation of the RTK [106]. The consequence of RTK activation, is the successive recruitment and activation of PI3K, AKT, and hundreds of target proteins that drive increased cell growth, metabolism, survival, and proliferation [106].

**Figure 4:**
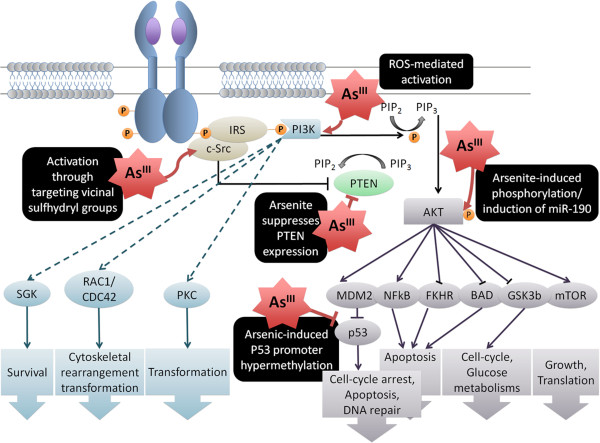
**Arsenic-mediated disruption of PI3K/AKT signaling pathway. **Depending on the receptor, different proteins can bind to the phosphorylated tyrosine residue of the RTK to recruit PI3K to the plasma membrane. There, the activated PI3K can interact with phosphatidylinositol 4,5-bisphosphate (PIP2) on the inner side of the membrane, and catalyze its phosphorylation to phosphatidylinositol 3,4,5-triphosphate (PIP3). PIP3 activates the kinase AKT, which is capable of phosphorylating a number of target proteins in the cytoplasm and nucleus. Some of the direct targets of PI3K (light blue) and AKT (grey), and their consequences on cell fate are depicted. Arsenic targets sulfhydryl groups of PI3K kinases such as c-Src, also resulting in activation of the PI3K/AKT pathway. As^III ^can also activate AKT independently of PI3K, both through STAT3 and/or induction of miR-190. PTEN is an inhibitor of the pathway that has been shown to be a target of arsenic in stem cells. Among other mechanisms, methylation patterns at the promoter region of the p53 gene have been shown to be modified by arsenic, resulting in silencing of this tumor suppressor.

Acute exposure to arsenite can stimulate the PI3K/AKT phosphorylation cascade, leading to cellular transformation characterized by increased proliferation and anchorage-independent growth [107-109] (Figure 4). As^III^ can induce phosphorylation of EZH2 at serine 21 in human bronchial epithelial cells and such phosphorylation of EZH2 requires As^III^-activated signalling through JNK and STAT3 leading to phosphorylation of AKT [110]. Arsenic-induced activation of AKT may be also associated with its ability to cause the induction of miR-190. This microRNA acts by repressing expression of the PH domain leucine-rich repeat protein phosphatase (PHLPP) - a negative regulator of AKT signaling [111]. Additionally, it has been shown that activation of the JNK-STAT3 pathway is involved in As^III^-induced AKT activation [102]. In HBECs, As^III^ can stimulate AKT and the consequent release of vascular endothelial growth factor (VEGF), inducing cell migration through different mechanisms [102,112,113]. During malignant transformation of stem cells, arsenite has also been shown to suppress expression of PTEN, an important inhibitor of PI3K/AKT signaling [114].

Although acute activation of this pathway is thought to be mediated by arsenic-induced ROS, the specific role of arsenic on PI3K/AKT signalling during chronic exposure remains to be clearly demonstrated [115].

### Arsenic and the Nrf2-KEAP1 signaling pathway

The transcription factor nuclear factor erythroid-derived factor 2–related factor 2 (NRF2) plays a key role in the activation of oxidative stress response. NRF2 contains a leucine-zipper DNA binding domain capable of binding to both antioxidant response elements (ARE’s) and electrophile response elements (ERE’s). Under normal conditions, NRF2 is actively sequestered by KEAP1 and targeted for proteolytic degradation [116]; however, under conditions of oxidative or chemical stress, NRF2 dissociates from KEAP1 and migrates to the nucleus to initiate a stress-related response. The KEAP1 E3-ubiquitin ligase complex is frequently affected by genetic disruption and aberrant expression in non-small cell lung cancer, resulting in NF-κB activation, is characteristic of lung tumorigenesis [117].

**Figure 5:**
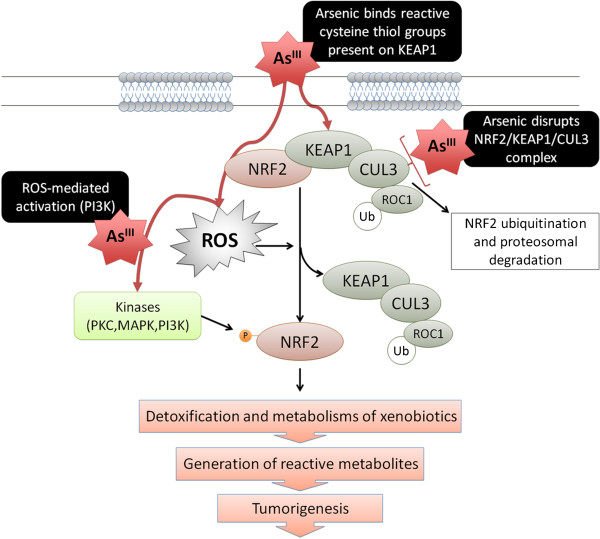
**Arsenic affects NRF2 signaling pathway. **The transcription factor nuclear factor erythroid-derived factor 2–related factor 2 (NRF2) has classically been associated with a cancer preventive function through the induction of cytoprotective proteins that inactivate reactive carcinogenic species and their intermediates. Arsenic can stimulate the activity of the NFR2 pathway mainly through generation of ROS. These events can protect the cell during acute/short-term exposure to low doses of arsenic. However, chronic arsenic-mediated activation of the NRF2 pathway may result in detrimental cellular effects associated with arsenic-induced pathogenesis.

It has been proposed that activation of the NRF2 pathway confers protection against toxic effects induced by both As^III^ and MMA^III^[118]. Pathological alterations in lung tissue, such as lung inflammatory response, induced by short-term exposure to arsenic can be prevented by NRF2 activation [119]. Arsenite can also stabilize NRF2 by disrupting the NRF2-KEAP1-CUL3 complex (Figure 5) [113]. It is possible that this occurs through the interaction of arsenic with KEAP1, since it has been reported that arsenic is capable of binding to reactive cysteine thiol groups present on KEAP1, thus triggering the dissociation of the complex and inducing constitutive NRF2-dependent signaling [120]. This apparent protective effect of NRF2 against arsenic toxicity has been observed most often at low doses; however, chronic low-dose exposure may overwhelm the arsenic-mediated NRF2-dependent protection, resulting in over-stimulation of NRF2-dependant genes [121].

## Conclusion and future directions

Lung cancer is the leading cause of cancer-related deaths in North America, affecting over 200,000 men and women each year [122]. Arsenic poisoning through contaminated drinking water leading to arsenic-induced lung cancer is a major public health concern; consequently, the mechanisms underlying the carcinogenic effects of arsenic in lung cancer has become an important avenue of research.

Undoubtedly, the biotransformation of As^V^ into As^III^ and its methylated conjugates plays a crucial role in arsenic carcinogenicity at both genetic and epigenetic levels. Genetic changes are acquired mainly through the induction of ROS during the biotransformation process, while the competition for methyl groups between As^V^ detoxification enzymes and DMT’s contribute to epigenetic abnormalities.

Arsenic species directly modulate several oncogenic pathways - most notably the EGFR, PI3K/AKT and the NRF2/KEAP1 pathways - and these specific pathways possess actionable targets for therapy in lung cancer. A greater understanding of the molecular mechanisms governing arsenic-related lung tumorigenesis may therefore yield promising translatable findings. Deep characterization of arsenic-related tumors and/or cell models at both the genetic and epigenetic levels, and the comparison of arsenic-related and unrelated SqCC tumors may provide such insights. On the other hand, mechanisms associated with anti-tumoral effects of As2O3 in the treatment of APL (not discussed in this review) should also be considered in order to increase the understanding of the molecular effects of arsenic in the human body.

In conclusion, arsenic can induce specific alterations affecting pathways that drive malignant transformation in lung cells. Current evidence suggests that arsenic-induced lung tumors represent a unique class of lung cancer, based on histology and underlying molecular characteristics. Further characterization of the mechanisms by which arsenic affects its targets will certainly give support to preventing and/or reducing the effects of arsenic toxicity, especially among those populations chronically exposed to arsenic.

## Abbreviations

AsIII: Arsenite; AsV: Arsenate; EGFR: Epidermal Growth Factor; HBEC: Human Bronchial Epithelial Cells; MMAIII: Monomethylarsonous Acid; NRF2: NFE2-Related Factor 2; PIK3: Phosphatidylinositol 3-kinase; ROS: Reactive Oxygen Species; RTK: Receptor Tyrosine Kinase; SAM: S-Adenosyl Methionine; SCC: Small Cell Carcinomas; SqCC: Squamous Cell Carcinomas.

## Competing interests

All authors declare no conflict of interest on the topics covered by this review.

## Authors’ contributions

RH and DBS contributed to manuscript conception and writing. KE and DR contributed to literature search and manuscript writing. SL and WLL contributed to manuscript writing and critically revised the paper. All authors read and approved the final manuscript. VM contributed to study conception, manuscript writing and critically revised the paper.

## References

[B1] IARC Some drinking-water disinfectants and contaminants, including arsenic. Monographs on chloramine, chloral and chloral hydrate, dichloroacetic acid, trichloroacetic acid and 3-chloro-4-(dichloromethyl)-5-hydroxy-2(5H)-furanone IARC Monogr Eval Carcinog Risks Hum 2004 84 269 477 15645578 PMC5220262

[B2] Smedley PL Kinniburgh DG A review of the source, behaviour and distribution of arsenic in natural waters Appl Geochem 2002 17 517 568 10.1016/S0883-2927(02)00018-5

[B3] Smith AH Lingas EO Mahfuzar R Contamination of drinking-water by arsenic in bangladesh: a public health emergency Bull World Health Organ 2000 78 1093 1103 11019458 PMC2560840

[B4] Putila JJ Guo NL Association of arsenic exposure with lung cancer incidence rates in the united states PLoS One 2011 6 e25886 10.1371/journal.pone.0025886 22003413 PMC3189216

[B5] U. S. Environmental Protection Agency National primary drinking water regulations; arsenic and clarifications to compliance and New source contaminants monitoring; final rule Book national primary drinking water regulations; arsenic and clarifications to compliance and New source contaminants monitoring; final rule vol. 66 2001 6975

[B6] Kumar A Adak P Gurian PL Lockwood JR Arsenic exposure in US public and domestic drinking water supplies: a comparative risk assessment J Expo Sci Environ Epidemiol 2010 20 245 254 10.1038/jes.2009.24 19401722

[B7] Nieder AM MacKinnon JA Fleming LE Kearney G Hu JJ Sherman RL Huang Y Lee DJ Bladder cancer clusters in florida: identifying populations at risk J Urol 2009 182 46 50 discussion 51 10.1016/j.juro.2009.02.149 19450849

[B8] Iland HJ Seymour JF Role of arsenic trioxide in acute promyelocytic leukemia Curr Treat Options Oncol 2013 [Epub ahead of print] 10.1007/s11864-012-0223-3 23322117

[B9] Mi J Current treatment strategy of acute promyelocytic leukemia Frontiers of medicine 2011 5 341 347 10.1007/s11684-011-0169-z 22198746

[B10] McGuigan CF Hamula CLA Huang S Gabos S Le XC A review on arsenic concentrations in canadian drinking water Environmental Reviews 2010 18 291 307 10.1139/A10-012

[B11] Ryker SJ Mapping arsenic in groundwater Geotimes 2001 46 34 36

[B12] Nordstrom DK Public health. Worldwide occurrences of arsenic in ground water Science 2002 296 2143 2145 10.1126/science.1072375 12077387

[B13] Smith AH Hopenhayn-Rich C Bates MN Goeden HM Hertz-Picciotto I Duggan HM Wood R Kosnett MJ Smith MT Cancer risks from arsenic in drinking water Environ Health Perspect 1992 97 259 267 1396465 10.1289/ehp.9297259 PMC1519547

[B14] Mead MN Arsenic: in search of an antidote to a global poison Environ Health Perspect 2005 113 A378 386 10.1289/ehp.113-a378 15929879 PMC1257621

[B15] Guo HR Wang NS Hu H Monson RR Cell type specificity of lung cancer associated with arsenic ingestion Cancer epidemiology, biomarkers & prevention: a publication of the American Association for Cancer Research, cosponsored by the American Society of Preventive Oncology 2004 13 638 643 15066930

[B16] Barrett JC Lamb PW Wiseman RW Multiple mechanisms for the carcinogenic effects of asbestos and other mineral fibers Environ Health Perspect 1989 81 81 89 2667990 10.1289/ehp.898181 PMC1567531

[B17] Ebert F Weiss A Bultemeyer M Hamann I Hartwig A Schwerdtle T Arsenicals affect base excision repair by several mechanisms Mutat Res 2011 715 32 41 10.1016/j.mrfmmm.2011.07.004 21782832

[B18] Pomroy C Charbonneau SM McCullough RS Tam GK Human retention studies with 74As Toxicol Appl Pharmacol 1980 53 550 556 10.1016/0041-008X(80)90368-3 7385250

[B19] Wang Y Fang J Leonard SS Rao KM Cadmium inhibits the electron transfer chain and induces reactive oxygen species Free Radic Biol Med 2004 36 1434 1443 10.1016/j.freeradbiomed.2004.03.010 15135180

[B20] Dilda PJ Hogg PJ Arsenical-based cancer drugs Cancer Treat Rev 2007 33 542 564 10.1016/j.ctrv.2007.05.001 17624680

[B21] Nemeti B Regonesi ME Tortora P Gregus Z Polynucleotide phosphorylase and mitochondrial ATP synthase mediate reduction of arsenate to the more toxic arsenite by forming arsenylated analogues of ADP and ATP Toxicological sciences: an official journal of the Society of Toxicology 2010 117 270 281 10.1093/toxsci/kfq141 20457661

[B22] Kala SV Neely MW Kala G Prater CI Atwood DW Rice JS Lieberman MW The MRP2/cMOAT transporter and arsenic-glutathione complex formation are required for biliary excretion of arsenic J Biol Chem 2000 275 33404 33408 10.1074/jbc.M007030200 10938093

[B23] Cullen WR Reimer KJ Arsenic speciation in the environment Chem Rev 1989 89 713 10.1021/cr00094a002

[B24] Styblo M Drobna Z Jaspers I Lin S Thomas DJ The role of biomethyl-ation in toxicity and carcinogenicity of arsenic: a research update Environ Health Persp 2002 110 767 10.1289/ehp.02110s5767 PMC1241242 12426129

[B25] Thomas DJ Styblo M Lin S The cellular metabolism and systemic toxicity of arsenic Toxicol Appl Pharmacol 2001 176 127 144 10.1006/taap.2001.9258 11601889

[B26] Klein CB Leszczynska J Hickey C Rossman TG Further evidence against a direct genotoxic mode of action for arsenic-induced cancer Toxicol Appl Pharmacol 2007 222 289 297 10.1016/j.taap.2006.12.033 17316729 PMC1986829

[B27] Kligerman AD Doerr CL Tennant AH Harrington-Brock K Allen JW Winkfield E Poorman-Allen P Kundu B Funasaka K Roop BC Methylated trivalent arsenicals as candidate ultimate genotoxic forms of arsenic: induction of chromosomal mutations but not gene mutations Environ Mol Mutagen 2003 42 192 205 10.1002/em.10192 14556226

[B28] Rossman TG Klein CB Genetic and epigenetic effects of environmental arsenicals Metallomics: integrated biometal science 2011 3 1135 1141 10.1039/c1mt00074h 21976018

[B29] Rossman TG Uddin AN Burns FJ Evidence that arsenite acts as a cocarcinogen in skin cancer Toxicol Appl Pharmacol 2004 198 394 404 10.1016/j.taap.2003.10.016 15276419

[B30] Hartwig A Groblinghoff UD Beyersmann D Natarajan AT Filon R Mullenders LH Interaction of arsenic(III) with nucleotide excision repair in UV-irradiated human fibroblasts Carcinogenesis 1997 18 399 405 10.1093/carcin/18.2.399 9054635

[B31] Jha AN Noditi M Nilsson R Natarajan AT Genotoxic effects of sodium arsenite on human cells Mutat Res 1992 284 215 221 10.1016/0027-5107(92)90005-M 1281272

[B32] Wiencke JK Yager JW Specificity of arsenite in potentiating cytogenetic damage induced by the DNA crosslinking agent diepoxybutane Environ Mol Mutagen 1992 19 195 200 10.1002/em.2850190303 1572342

[B33] Li JH Rossman TG Mechanism of comutagenesis of sodium arsenite with n-methyl-n-nitrosourea Biol Trace Elem Res 1989 21 373 381 10.1007/BF02917278 2484616

[B34] Lee TC Huang RY Jan KY Sodium arsenite enhances the cytotoxicity, clastogenicity, and 6-thioguanine-resistant mutagenicity of ultraviolet light in chinese hamster ovary cells Mutat Res 1985 148 83 89 10.1016/0027-5107(85)90210-6 3969080

[B35] Flora SJ Arsenic-induced oxidative stress and its reversibility Free Radic Biol Med 2011 51 257 281 10.1016/j.freeradbiomed.2011.04.008 21554949

[B36] Rossman TG Mechanism of arsenic carcinogenesis: an integrated approach Mutat Res 2003 533 37 65 10.1016/j.mrfmmm.2003.07.009 14643412

[B37] Naranmandura H Xu S Sawata T Hao WH Liu H Bu N Ogra Y Lou YJ Suzuki N Mitochondria are the main target organelle for trivalent monomethylarsonous acid (MMA(III))-induced cytotoxicity Chem Res Toxicol 2011 24 1094 1103 10.1021/tx200156k 21648415

[B38] Turrens JF Superoxide production by the mitochondrial respiratory chain Biosci Rep 1997 17 3 8 10.1023/A:1027374931887 9171915

[B39] Kitchin KT Wallace K Evidence against the nuclear in situ binding of arsenicals–oxidative stress theory of arsenic carcinogenesis Toxicol Appl Pharmacol 2008 232 252 257 10.1016/j.taap.2008.06.021 18671993

[B40] Halliwell B Oxidative stress and cancer: have we moved forward? Biochem J 2007 401 1 11 17150040 10.1042/BJ20061131

[B41] Martinez VD Vucic EA Becker-Santos DD Gil L Lam WL Arsenic exposure and the induction of human cancers J Toxicol 2011 2011 431287 22174709 10.1155/2011/431287 PMC3235889

[B42] Gurr J-R Yih L-H Samikkannu T Bau D-T Lin S-Y Jan K-Y Nitric oxide production by arsenite Mutation Research/Fundamental and Molecular Mechanisms of Mutagenesis 2003 533 173 182 10.1016/j.mrfmmm.2003.08.024 14643419

[B43] Wink DA Kasprzak KS Maragos CM Elespuru RK Misra M Dunams TM Cebula TA Koch WH Andrews AW Allen JS DNA deaminating ability and genotoxicity of nitric oxide and its progenitors Science 1991 254 1001 1003 10.1126/science.1948068 1948068

[B44] Radi R Beckman JS Bush KM Freeman BA Peroxynitrite oxidation of sulfhydryls The cytotoxic potential of superoxide and nitric oxide. The Journal of biological chemistry 1991 266 4244 4250 1847917

[B45] Leaf CD Wishnok JS Tannenbaum SR Endogenous incorporation of nitric oxide from L-arginine into N-nitrosomorpholine stimulated by escherichia coli lipopolysaccharide in the rat Carcinogenesis 1991 12 537 539 10.1093/carcin/12.3.537 1849054

[B46] Tsuda M Kurashima Y Tobacco smoking, chewing, and snuff dipping: factors contributing to the endogenous formation of N-nitroso compounds Crit Rev Toxicol 1991 21 243 253 10.3109/10408449109017912 2069710

[B47] Beckman JS Beckman TW Chen J Marshall PA Freeman BA Apparent hydroxyl radical production by peroxynitrite: implications for endothelial injury from nitric oxide and superoxide Proc Natl Acad Sci USA 1990 87 1620 1624 10.1073/pnas.87.4.1620 2154753 PMC53527

[B48] Andrew AS Karagas MR Hamilton JW Decreased DNA repair gene expression among individuals exposed to arsenic in united states drinking water Int J Cancer 2003 104 263 268 10.1002/ijc.10968 12569548

[B49] Andrew AS Burgess JL Meza MM Demidenko E Waugh MG Hamilton JW Karagas MR Arsenic exposure is associated with decreased DNA repair in vitro and in individuals exposed to drinking water arsenic Environ Health Perspect 2006 114 1193 1198 10.1289/ehp.9008 16882524 PMC1552016

[B50] Nollen M Ebert F Moser J Mullenders LH Hartwig A Schwerdtle T Impact of arsenic on nucleotide excision repair: XPC function, protein level, and gene expression Mol Nutr Food Res 2009 53 572 582 10.1002/mnfr.200800480 19382146

[B51] Walter I Schwerdtle T Thuy C Parsons JL Dianov GL Hartwig A Impact of arsenite and its methylated metabolites on PARP-1 activity, PARP-1 gene expression and poly(ADP-ribosyl)ation in cultured human cells DNA Repair 2007 6 61 70 10.1016/j.dnarep.2006.08.008 17011244

[B52] Osmond MJ Kunz BA Snow ET Age and exposure to arsenic alter base excision repair transcript levels in mice Mutagenesis 2010 25 517 522 10.1093/mutage/geq037 20643705

[B53] Wen G Calaf GM Partridge MA Echiburu-Chau C Zhao Y Huang S Chai Y Li B Hu B Hei TK Neoplastic transformation of human small airway epithelial cells induced by arsenic Mol Med 2008 14 2 10 18037969 10.2119/2007-00090.Wen PMC2082132

[B54] Zhao Y Toselli P Li W Microtubules as a critical target for arsenic toxicity in lung cells in vitro and in vivo Int J Environ Res Public Health 2012 9 474 495 10.3390/ijerph9020474 22470304 PMC3315258

[B55] Sciandrello G Caradonna F Mauro M Barbata G Arsenic-induced DNA hypomethylation affects chromosomal instability in mammalian cells Carcinogenesis 2004 25 413 417 14633664 10.1093/carcin/bgh029

[B56] Sciandrello G Barbaro R Caradonna F Barbata G Early induction of genetic instability and apoptosis by arsenic in cultured chinese hamster cells Mutagenesis 2002 17 99 103 10.1093/mutage/17.2.99 11880537

[B57] Vega L Gonsebatt ME Ostrosky-Wegman P Aneugenic effect of sodium arsenite on human lymphocytes in vitro: an individual susceptibility effect detected Mutat Res 1995 334 365 373 10.1016/0165-1161(95)90074-8 7753100

[B58] Vogt BL Rossman TG Effects of arsenite on p53, p21 and cyclin D expression in normal human fibroblasts — a possible mechanism for arsenite’s comutagenicity Mutation Research/Fundamental and Molecular Mechanisms of Mutagenesis 2001 478 159 168 10.1016/S0027-5107(01)00137-3 11406180

[B59] Tang F Liu G He Z Ma WY Bode AM Dong Z Arsenite inhibits p53 phosphorylation, DNA binding activity, and p53 target gene p21 expression in mouse epidermal JB6 cells Mol Carcinog 2006 45 861 870 10.1002/mc.20245 16739126

[B60] Huang Y Zhang J McHenry KT Kim MM Zeng W Lopez-Pajares V Dibble CC Mizgerd JP Yuan ZM Induction of cytoplasmic accumulation of p53: a mechanism for low levels of arsenic exposure to predispose cells for malignant transformation Cancer Res 2008 68 9131 9136 10.1158/0008-5472.CAN-08-3025 19010883 PMC2717853

[B61] Komissarova EV Rossman TG Arsenite induced poly(ADP-ribosyl)ation of tumor suppressor P53 in human skin keratinocytes as a possible mechanism for carcinogenesis associated with arsenic exposure Toxicol Appl Pharmacol 2010 243 399 404 10.1016/j.taap.2009.12.014 20036271 PMC2830301

[B62] Qin XJ Liu W Li YN Sun X Hai CX Hudson LG Liu KJ Poly(ADP-ribose) polymerase-1 inhibition by arsenite promotes the survival of cells with unrepaired DNA lesions induced by UV exposure Toxicological sciences: an official journal of the Society of Toxicology 2012 127 120 129 10.1093/toxsci/kfs099 22387748 PMC3327874

[B63] Martinez VD Buys TP Adonis M Benitez H Gallegos I Lam S Lam WL Gil L Arsenic-related DNA copy-number alterations in lung squamous cell carcinomas Br J Cancer 2010 103 1277 1283 10.1038/sj.bjc.6605879 20842114 PMC2967055

[B64] Tonon G Wong KK Maulik G Brennan C Feng B Zhang Y Khatry DB Protopopov A You MJ Aguirre AJ High-resolution genomic profiles of human lung cancer Proc Natl Acad Sci USA 2005 102 9625 9630 10.1073/pnas.0504126102 15983384 PMC1160520

[B65] Venkatesan RN Treuting PM Fuller ED Goldsby RE Norwood TH Gooley TA Ladiges WC Preston BD Loeb LA Mutation at the polymerase active site of mouse DNA polymerase delta increases genomic instability and accelerates tumorigenesis Mol Cell Biol 2007 27 7669 7682 10.1128/MCB.00002-07 17785453 PMC2169052

[B66] Parsons JL Preston BD O'Connor TR Dianov GL DNA polymerase delta-dependent repair of DNA single strand breaks containing 3'-end proximal lesions Nucleic Acids Res 2007 35 1054 1063 10.1093/nar/gkl1115 17264132 PMC1851633

[B67] Goldsby RE Hays LE Chen X Olmsted EA Slayton WB Spangrude GJ Preston BD High incidence of epithelial cancers in mice deficient for DNA polymerase delta proofreading Proc Natl Acad Sci USA 2002 99 15560 15565 10.1073/pnas.232340999 12429860 PMC137756

[B68] Simeonova PP Luster MI Mechanisms of arsenic carcinogenicity: genetic or epigenetic mechanisms? J Environ Pathol Toxicol Oncol 2000 19 281 286 10983894

[B69] Mazumder DN Effect of chronic intake of arsenic-contaminated water on liver Toxicol Appl Pharmacol 2005 206 169 175 10.1016/j.taap.2004.08.025 15967205

[B70] Tseng CH Chong CK Chen CJ Tai TY Dose–response relationship between peripheral vascular disease and ingested inorganic arsenic among residents in blackfoot disease endemic villages in taiwan Atherosclerosis 1996 120 125 133 10.1016/0021-9150(95)05693-9 8645353

[B71] Engel RR Hopenhayn-Rich C Receveur O Smith AH Vascular effects of chronic arsenic exposure: a review Epidemiol Rev 1994 16 184 209 7713176 10.1093/oxfordjournals.epirev.a036150

[B72] Intarasunanont P Navasumrit P Woraprasit S Chaisatra K Suk WA Mahidol C Ruchirawat M Effects of arsenic exposure on DNA methylation in cord blood samples from newborn babies and in a human lymphoblast cell line Environmental health: a global access science source 2012 11 31 22551203 10.1186/1476-069X-11-31 PMC3506565

[B73] Jensen TJ Wozniak RJ Eblin KE Wnek SM Gandolfi AJ Futscher BW Epigenetic mediated transcriptional activation of WNT5A participates in arsenical-associated malignant transformation Toxicol Appl Pharmacol 2009 235 39 46 10.1016/j.taap.2008.10.013 19061910 PMC4438681

[B74] Reichard JF Puga A Effects of arsenic exposure on DNA methylation and epigenetic gene regulation Epigenomics 2010 2 87 104 10.2217/epi.09.45 20514360 PMC2877392

[B75] Cui X Wakai T Shirai Y Hatakeyama K Hirano S Chronic oral exposure to inorganic arsenate interferes with methylation status of p16INK4a and RASSF1A and induces lung cancer in a/J mice Toxicological sciences: an official journal of the Society of Toxicology 2006 91 372 381 10.1093/toxsci/kfj159 16543296

[B76] Marsit CJ Eddy K Kelsey KT MicroRNA responses to cellular stress Cancer Res 2006 66 10843 10848 10.1158/0008-5472.CAN-06-1894 17108120

[B77] Zhao CQ Young MR Diwan BA Coogan TP Waalkes MP Association of arsenic-induced malignant transformation with DNA hypomethylation and aberrant gene expression Proc Natl Acad Sci USA 1997 94 10907 10912 10.1073/pnas.94.20.10907 9380733 PMC23527

[B78] Mass MJ Wang L Arsenic alters cytosine methylation patterns of the promoter of the tumor suppressor gene p53 in human lung cells: a model for a mechanism of carcinogenesis Mutat Res 1997 386 263 277 10.1016/S1383-5742(97)00008-2 9219564

[B79] Chiang PK Gordon RK Tal J Zeng GC Doctor BP Pardhasaradhi K McCann PP S-adenosylmethionine and methylation FASEB journal: official publication of the Federation of American Societies for Experimental Biology 1996 10 471 480 8647346

[B80] Jensen TJ Novak P Eblin KE Gandolfi AJ Futscher BW Epigenetic remodeling during arsenical-induced malignant transformation Carcinogenesis 2008 29 1500 1508 10.1093/carcin/bgn102 18448484 PMC2516486

[B81] Ren X McHale CM Skibola CF Smith AH Smith MT Zhang L An emerging role for epigenetic dysregulation in arsenic toxicity and carcinogenesis Environ Health Perspect 2011 119 11 19 20682481 10.1289/ehp.1002114 PMC3018488

[B82] Salnikow K Zhitkovich A Genetic and epigenetic mechanisms in metal carcinogenesis and cocarcinogenesis: nickel, arsenic, and chromium Chem Res Toxicol 2008 21 28 44 10.1021/tx700198a 17970581 PMC2602826

[B83] Loenen WA S-adenosylmethionine: jack of all trades and master of everything? Biochem Soc Trans 2006 34 330 333 16545107 10.1042/BST20060330

[B84] Chen WT Hung WC Kang WY Huang YC Chai CY Urothelial carcinomas arising in arsenic-contaminated areas are associated with hypermethylation of the gene promoter of the death-associated protein kinase Histopathology 2007 51 785 792 10.1111/j.1365-2559.2007.02871.x 17953697

[B85] Chai CY Huang YC Hung WC Kang WY Chen WT Arsenic salts induced autophagic cell death and hypermethylation of DAPK promoter in SV-40 immortalized human uroepithelial cells Toxicol Lett 2007 173 48 56 10.1016/j.toxlet.2007.06.006 17683884

[B86] Vogt BL Rossman TG Effects of arsenite on p53, p21 and cyclin D expression in normal human fibroblasts – a possible mechanism for arsenite’s comutagenicity Mutat Res 2001 478 159 168 10.1016/S0027-5107(01)00137-3 11406180

[B87] Ziech D Franco R Pappa A Panayiotidis MI Reactive oxygen species (ROS)–induced genetic and epigenetic alterations in human carcinogenesis Mutat Res 2011 711 167 173 10.1016/j.mrfmmm.2011.02.015 21419141

[B88] Chanda S Dasgupta UB Guhamazumder D Gupta M Chaudhuri U Lahiri S Das S Ghosh N Chatterjee D DNA hypermethylation of promoter of gene p53 and p16 in arsenic-exposed people with and without malignancy Toxicological sciences: an official journal of the Society of Toxicology 2006 89 431 437 10.1093/toxsci/kfj030 16251483

[B89] Jo WJ Ren X Chu F Aleshin M Wintz H Burlingame A Smith MT Vulpe CD Zhang L Acetylated H4K16 by MYST1 protects UROtsa cells from arsenic toxicity and is decreased following chronic arsenic exposure Toxicol Appl Pharmacol 2009 241 294 302 10.1016/j.taap.2009.08.027 19732783 PMC2784148

[B90] Zhou X Sun H Ellen TP Chen H Costa M Arsenite alters global histone H3 methylation Carcinogenesis 2008 29 1831 1836 10.1093/carcin/bgn063 18321869 PMC2722848

[B91] Wang Z Zhao Y Smith E Goodall GJ Drew PA Brabletz T Yang C Reversal and prevention of arsenic-induced human bronchial epithelial cell malignant transformation by microRNA-200b Toxicological sciences: an official journal of the Society of Toxicology 2011 121 110 122 10.1093/toxsci/kfr029 21292642 PMC3080188

[B92] Cui Y Han Z Hu Y Song G Hao C Xia H Ma X MicroRNA-181b and microRNA-9 mediate arsenic-induced angiogenesis via NRP1 J Cell Physiol 2012 227 772 783 10.1002/jcp.22789 21503876

[B93] Yarden Y Sliwkowski MX Untangling the ErbB signalling network Nat Rev Mol Cell Biol 2001 2 127 137 10.1038/35052073 11252954

[B94] Andrew AS Mason RA Memoli V Duell EJ Arsenic activates EGFR pathway signaling in the lung Toxicological sciences: an official journal of the Society of Toxicology 2009 109 350 357 10.1093/toxsci/kfp015 19168569 PMC2683921

[B95] Sung TI Wang YJ Chen CY Hung TL Guo HR Increased serum level of epidermal growth factor receptor in liver cancer patients and its association with exposure to arsenic Sci Total Environ 2012 424 74 78 22446113 10.1016/j.scitotenv.2012.02.079

[B96] Biscardi JS Maa MC Tice DA Cox ME Leu TH Parsons SJ c-Src-mediated phosphorylation of the epidermal growth factor receptor on Tyr845 and Tyr1101 is associated with modulation of receptor function J Biol Chem 1999 274 8335 8343 10.1074/jbc.274.12.8335 10075741

[B97] Tice DA Biscardi JS Nickles AL Parsons SJ Mechanism of biological synergy between cellular Src and epidermal growth factor receptor Proc Natl Acad Sci USA 1999 96 1415 1420 10.1073/pnas.96.4.1415 9990038 PMC15477

[B98] Simeonova PP Luster MI Arsenic carcinogenicity: relevance of c-Src activation Mol Cell Biochem 2002 234–235 277 282 12162444

[B99] Li G Lee LS Li M Tsao SW Chiu JF Molecular changes during arsenic-induced cell transformation J Cell Physiol 2011 226 3225 3232 10.1002/jcp.22683 21344382

[B100] Liu LZ Jiang Y Carpenter RL Jing Y Peiper SC Jiang BH Role and mechanism of arsenic in regulating angiogenesis PLoS One 2011 6 e20858 10.1371/journal.pone.0020858 21687637 PMC3110823

[B101] Cheng HY Li P David M Smithgall TE Feng L Lieberman MW Arsenic inhibition of the JAK-STAT pathway Oncogene 2004 23 3603 3612 10.1038/sj.onc.1207466 15116095

[B102] Liu J Chen B Lu Y Guan Y Chen F JNK-dependent Stat3 phosphorylation contributes to Akt activation in response to arsenic exposure Toxicological sciences: an official journal of the Society of Toxicology 2012 129 363 371 10.1093/toxsci/kfs199 22696236 PMC3529643

[B103] Herbert KJ Snow ET Modulation of arsenic-induced epidermal growth factor receptor pathway signalling by resveratrol Chem Biol Interact 2012 198 38 48 10.1016/j.cbi.2012.05.004 22634503

[B104] Verma A Mohindru M Deb DK Sassano A Kambhampati S Ravandi F Minucci S Kalvakolanu DV Platanias LC Activation of Rac1 and the p38 mitogen-activated protein kinase pathway in response to arsenic trioxide J Biol Chem 2002 277 44988 44995 10.1074/jbc.M207176200 12239215

[B105] Cheng L Alexander RE Maclennan GT Cummings OW Montironi R Lopez-Beltran A Cramer HM Davidson DD Zhang S Molecular pathology of lung cancer: key to personalized medicine Modern pathology: an official journal of the United States and Canadian Academy of Pathology, Inc 2012 25 347 369 10.1038/modpathol.2011.215 22282308

[B106] Papadimitrakopoulou V Development of PI3K/AKT/mTOR pathway inhibitors and their application in personalized therapy for non-small-cell lung cancer Journal of thoracic oncology: official publication of the International Association for the Study of Lung Cancer 2012 7 1315 1326 10.1097/JTO.0b013e31825493eb 22648207

[B107] Stueckle TA Lu Y Davis ME Wang L Jiang BH Holaskova I Schafer R Barnett JB Rojanasakul Y Chronic occupational exposure to arsenic induces carcinogenic gene signaling networks and neoplastic transformation in human lung epithelial cells Toxicol Appl Pharmacol 2012 261 204 216 10.1016/j.taap.2012.04.003 22521957 PMC3358533

[B108] Gao N Shen L Zhang Z Leonard SS He H Zhang XG Shi X Jiang BH Arsenite induces HIF-1alpha and VEGF through PI3K, Akt and reactive oxygen species in DU145 human prostate carcinoma cells Mol Cell Biochem 2004 255 33 45 14971644 10.1023/b:mcbi.0000007259.65742.16

[B109] Dong Z The molecular mechanisms of arsenic-induced cell transformation and apoptosis Environ Health Perspect 2002 110 Suppl 5 757 759 12426127 10.1289/ehp.02110s5757 PMC1241240

[B110] Chen B Liu J Chang Q Beezhold K Lu Y Chen F JNK and STAT3 signaling pathways converge on Akt-mediated phosphorylation of EZH2 in bronchial epithelial cells induced by arsenic Cell Cycle 2012 12 10.4161/cc.23030 PMC3570498 23255093

[B111] Beezhold K Liu J Kan H Meighan T Castranova V Shi X Chen F miR-190-mediated downregulation of PHLPP contributes to arsenic-induced Akt activation and carcinogenesis Toxicological sciences: an official journal of the Society of Toxicology 2011 123 411 420 10.1093/toxsci/kfr188 21750348 PMC3179680

[B112] Wang Z Yang J Fisher T Xiao H Jiang Y Yang C Akt activation is responsible for enhanced migratory and invasive behavior of arsenic-transformed human bronchial epithelial cells Environ Health Perspect 2012 120 92 97 21954225 10.1289/ehp.1104061 PMC3261952

[B113] Zhang Y Bhatia D Xia H Castranova V Shi X Chen F Nucleolin links to arsenic-induced stabilization of GADD45alpha mRNA Nucleic Acids Res 2006 34 485 495 10.1093/nar/gkj459 16421274 PMC1342039

[B114] Tokar EJ Diwan BA Waalkes MP Arsenic exposure transforms human epithelial stem/progenitor cells into a cancer stem-like phenotype Environ Health Perspect 2010 118 108 115 20056578 10.1289/ehp.0901059 PMC2831952

[B115] Ling M Li Y Xu Y Pang Y Shen L Jiang R Zhao Y Yang X Zhang J Zhou J Regulation of miRNA-21 by reactive oxygen species-activated ERK/NF-kappaB in arsenite-induced cell transformation Free Radic Biol Med 2012 52 1508 1518 10.1016/j.freeradbiomed.2012.02.020 22387281

[B116] Zhang DD Mechanistic studies of the Nrf2-Keap1 signaling pathway Drug Metab Rev 2006 38 769 789 10.1080/03602530600971974 17145701

[B117] Thu KL Pikor LA Chari R Wilson IM Macaulay CE English JC Tsao MS Gazdar AF Lam S Lam WL Lockwood WW Genetic disruption of KEAP1/CUL3 E3 ubiquitin ligase complex components is a key mechanism of NF-kappaB pathway activation in lung cancer Journal of thoracic oncology: official publication of the International Association for the Study of Lung Cancer 2011 6 1521 1529 10.1097/JTO.0b013e3182289479 PMC3164321 21795997

[B118] Wang XJ Sun Z Chen W Eblin KE Gandolfi JA Zhang DD Nrf2 Protects human bladder urothelial cells from arsenite and monomethylarsonous acid toxicity Toxicol Appl Pharmacol 2007 225 206 213 10.1016/j.taap.2007.07.016 17765279 PMC2610476

[B119] Zheng Y Tao S Lian F Chau BT Chen J Sun G Fang D Lantz RC Zhang DD Sulforaphane prevents pulmonary damage in response to inhaled arsenic by activating the Nrf2-defense response Toxicol Appl Pharmacol 2012 265 292 299 10.1016/j.taap.2012.08.028 22975029 PMC3725323

[B120] Andujar P Wang J Descatha A Galateau-Salle F Abd-Alsamad I Billon-Galland MA Blons H Clin B Danel C Housset B p16INK4A Inactivation mechanisms in non-small-cell lung cancer patients occupationally exposed to asbestos Lung Cancer 2010 67 23 30 10.1016/j.lungcan.2009.03.018 19375815

[B121] Wang XJ Sun Z Chen W Li Y Villeneuve NF Zhang DD Activation of Nrf2 by arsenite and monomethylarsonous acid is independent of Keap1-C151: enhanced Keap1-Cul3 interaction Toxicol Appl Pharmacol 2008 230 383 389 10.1016/j.taap.2008.03.003 18417180 PMC2610481

[B122] American Cancer Society Cancer facts & figures 2012 Book cancer facts & figures 2012 2012 Atlanta: American Cancer Society

